# Massive Lumbar Disc Extrusion and Cauda Equina Syndrome in a Female Athlete Following Deadlift Training

**DOI:** 10.7759/cureus.100428

**Published:** 2025-12-30

**Authors:** Moath Alshanqity, Abdulrahman Y Sabbagh, Jannat A Abdulmuttalib

**Affiliations:** 1 Emergency Medicine, Ministry of National Guard Health Affairs, Jeddah, SAU; 2 Emergency Medicine, King Fahad Medical City, Riyadh, SAU; 3 Emergency Medicine, Riyadh Second Health Cluster, Riyadh, SAU; 4 Emergency Medicine, College of Medicine, Alfaisal University, Riyadh, SAU

**Keywords:** cauda equina syndrome, deadlift injury, emergency medicine, female athlete, lumbar disc extrusion, sports medicine

## Abstract

We present the case of a 26-year-old female bodybuilder who developed cauda equina syndrome (CES) following repetitive deadlift training. Her symptoms were initially misattributed to muscular strain, resulting in a delayed diagnosis. MRI revealed a massive L5-S1 disc extrusion compressing the cauda equina. She underwent emergency decompression and fusion with partial neurological recovery. This case highlights the importance of recognizing red-flag symptoms and early imaging in athletic populations.

## Introduction

Cauda equina syndrome (CES) is an unusual but potentially serious condition caused by the compression of nerve roots at the distal end of the spinal cord, leading to motor and sensory deficits in the lower extremities, as well as urinary, bowel, and sexual dysfunction [1]. According to a research study in Scotland, the crude rate of emergency spinal surgeries was 2.7 per 100,000 people per year, with women aged 30 to 39 having the highest rates [2]. In athletes, particularly those engaged in significant weightlifting activities such as deadlifting, incorrect technique or unsupervised training might increase the risk of back injuries [3]. Deadlifting puts a lot of stress on the lumbar spine, both compressive and shear. For women, the pressures are above 8 Kilonewton (kN), and for men, they are over 18 kN. This increases the likelihood of disc herniation and neurological compression [4]. We report a rare case of CES in a young female athlete caused by a massive lumbar disc extrusion following deadlift training.

## Case presentation

A 26-year-old female bodybuilder with no significant past medical history presented to the emergency department with a one-year history of worsening lower back pain associated with deadlift training. Initially diagnosed with a muscular strain and treated conservatively, she resumed training without supervision. However, her symptoms worsened over the past two weeks, with the development of left leg pain, weakness, and numbness. Additionally, she began experiencing vaginal and rectal paresthesia, although she did not report any incontinence.

**Figure 1:**
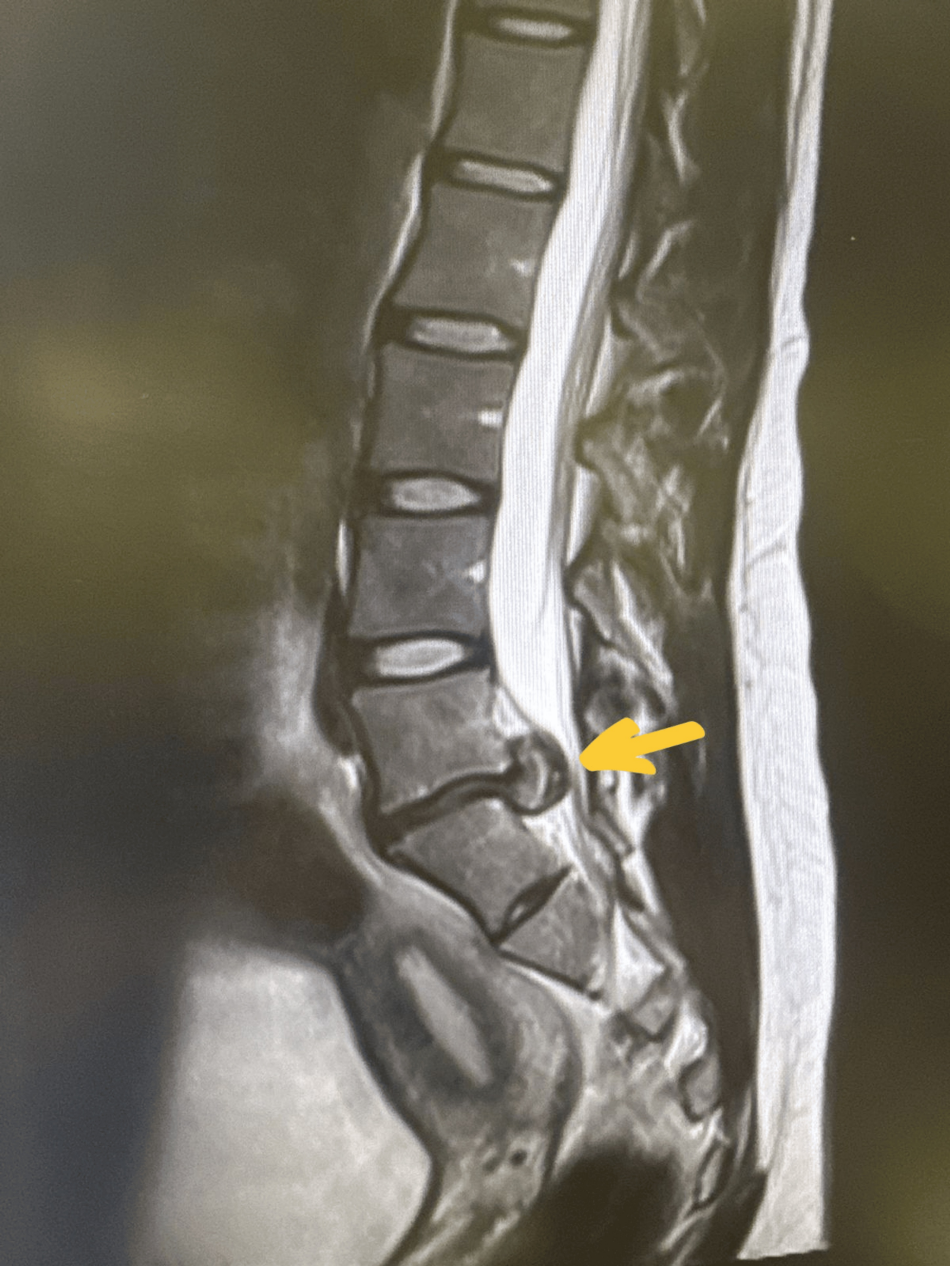
Sequential sagittal T2-weighted MRI image showing a massive central disc extrusion at L5–S1 with cranio-caudal migration (yellow arrow), causing severe compression of the cauda equina and bilateral S1 nerve roots.

**Figure 2:**
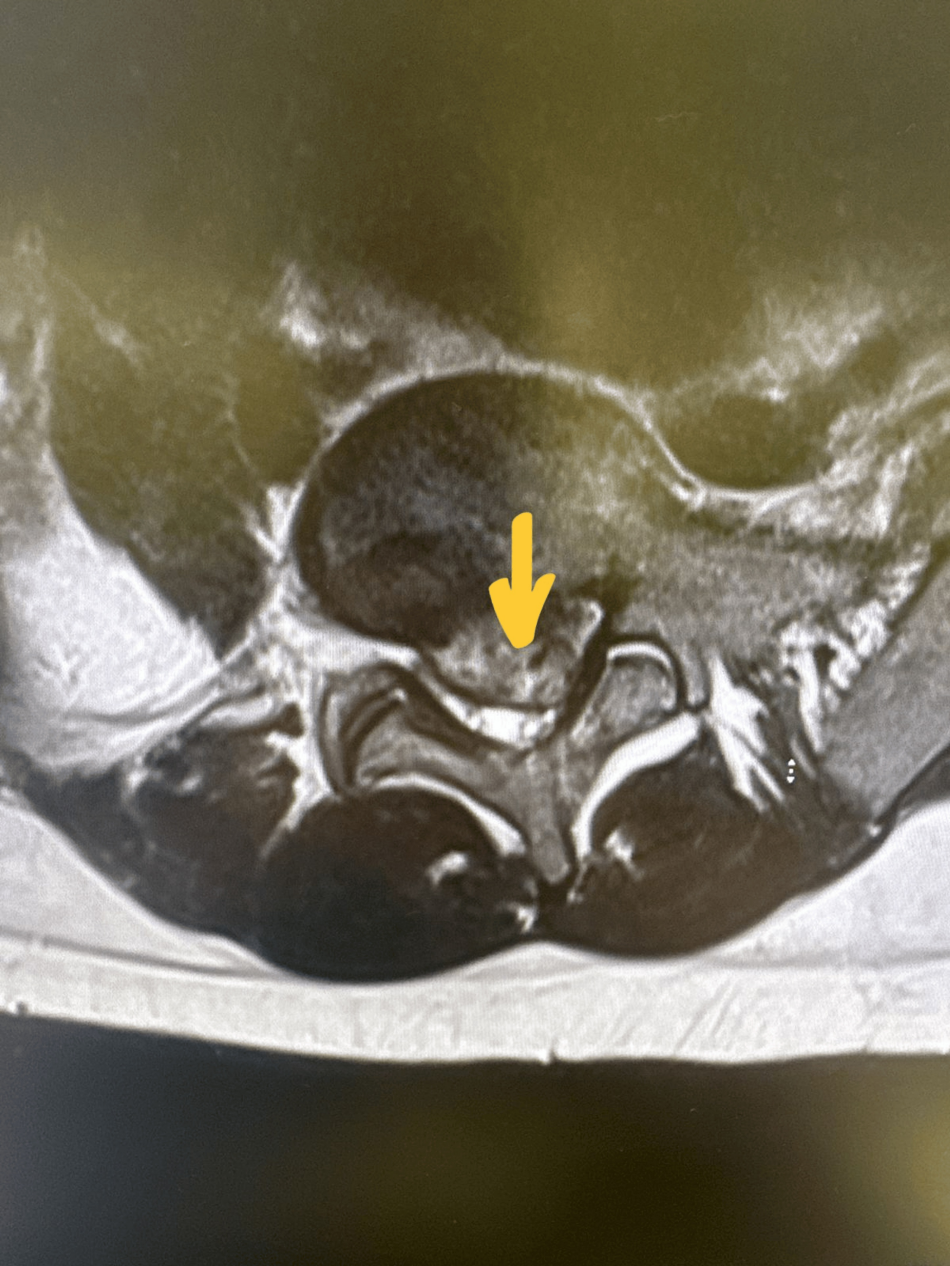
Sequential axial T2-weighted MRI image showing posterior disc protrusion (yellow arrow), causing severe spinal canal narrowing and bilateral recess stenosis.

Neurological examination revealed motor weakness in the left leg (L4: 4/5, L5: 3/5), decreased sensation in the L5-S1 dermatomes, hyporeflexia, and reduced rectal tone. MRI demonstrated a massive central and bilateral paracentral disc extrusion at L5-S1, with cranio-caudal migration, causing significant central stenosis and compression of the cauda equina roots, as well as severe compression of the traversing S1 nerve roots bilaterally (Figures 1, 2).

**Figure 3:**
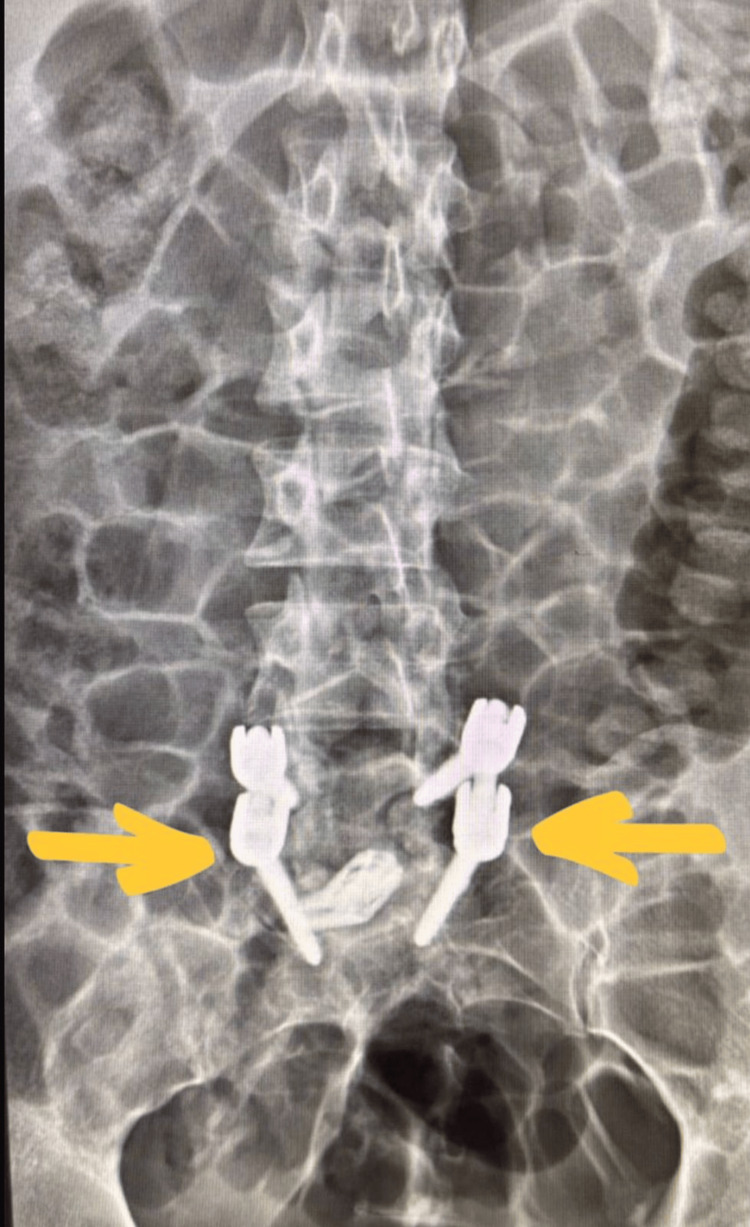
Postoperative anteroposterior X-ray showing pedicle screw fixation at L5–S1 following decompression and fusion.

Given the severity of the neurological deficits, the patient underwent emergency open transforaminal lumbar interbody fusion at L5-S1, which was performed successfully. Postoperative plain radiographs confirmed satisfactory positioning of the instrumentation at L5-S1 (Figure 3).

On the second day postoperatively, the patient showed significant improvement in motor function and sensation, and sphincter tone stabilized. However, mild residual numbness persisted in the left leg.

Following discharge, the patient was enrolled in a structured rehabilitation program including supervised physiotherapy and gradual return to activity. At three months postoperatively, she reported further neurological improvement and was able to resume supervised physical activities.

## Discussion

CES is a rare and possibly disabling condition that happens when the nerve roots at the bottom of the spinal cord are compressed. It often happens because of lumbar disc herniation, which can cause a lot of neurological damage, such as motor and sensory deficits in the lower extremities, as well as urinary, bowel, and sexual dysfunction [1]. In this case, a 26-year-old female bodybuilder got CES after a year of lower back pain that got worse while she was training for the deadlift. Heavy loads on the spine during deadlift training have been linked to strong compressive forces on the lumbar spine, which could damage discs and compress nerves [4]. Our case uniquely associates deadlifting with the CES, even though this link has not been reported before.

Rai et al. (2021) documented a case involving a 38-year-old male military member who developed CES following repetitive strenuous physical exertion during training [5]. The patient exhibited signs of CES accompanied by significant spinal stress, which is particularly relevant to our situation. Their case had immediate, severe symptoms, but our patient's symptoms got worse over the course of a year. This contrast highlights the diverse manifestations of CES, which may progress gradually, particularly in athletes who persist with rigorous training despite worsening symptoms.

Moussa et al. (2021) documented acute CES in a 15-year-old child after heavy lifting, signifying a significant L4-L5 disc herniation necessitating prompt decompression [6]. In their case, the symptoms got worse rapidly, but in our patient's case, they got worse more slowly, with a less dramatic drop in neurological function. This slow progression shows how important it is to identify the problem early, even if the symptoms come on slowly. Getting treatment early can make a big difference in how things turn out. In our case, emergency decompression surgery significantly enhanced motor function and sensation, underscoring the advantages of prompt surgical intervention, even in cases of progressive disease advancement.

Prior research on athletes with lumbar disc herniation has demonstrated that a significant proportion resume sporting activities post-therapy. About 78.9% of them come back after conservative treatments, and 85% come back after surgical microdiscectomy, usually within 4-6 months [7]. Despite the severity of CES, our patient got a lot of her motor function back, and by the time of the follow-up, she was able to do light physical activity with supervision, which is what has happened in other cases of lumbar spine pathology.

## Conclusions

This case underscores the susceptibility to CES among athletes, particularly those engaged in heavy lifting activities such as deadlifting. The fact that this patient's symptoms are getting worse over time shows how important it is to intervene quickly to protect the nervous system from long-term damage. Deadlifting requires a lot of strength; therefore, athletes need to be aware of the dangers of using an improper technique or training without supervision. Emergency doctors, sports doctors, and physiotherapists need to be very vigilant when assessing athletes who have neurological symptoms. A timely diagnosis and treatment can lead to better outcomes.
